# Foot lesions and forelimb skin abrasions in suckling piglets: development and risk factors

**DOI:** 10.1186/s40813-023-00351-9

**Published:** 2024-01-04

**Authors:** Marcus Heimann, Maria Hartmann, Fritjof Freise, Lothar Kreienbrock, Elisabeth grosse Beilage

**Affiliations:** 1 Field Station for Epidemiology, University of Veterinary Medicine Hannover, Buescheler Str. 9, 49456 Bakum, Germany; 2 Department of Biometry, Epidemiology and Information Processing, WHO Collaborating Centre for Research and Training for Health at the Human-Animal-Environment Interface, University of Veterinary Medicine Hannover, Buenteweg 2, 30559 Hannover, Germany

**Keywords:** Heel bruising, Sole bruising, Erosion, Floor, Animal welfare, SINS

## Abstract

**Background:**

Foot lesions in suckling piglets have been associated with poor flooring in several studies and were recently proposed to be indicative of swine inflammatory and necrosis syndrome. However, identical findings are also the typical outcome of various non-infectious causes; thus, further risk analysis is needed. The objective of this study was to describe the development of heel bruising, coronary band lesions and forelimb skin abrasion in suckling pigs up to 5 days of age. Furthermore, the effects of various intrinsic and extrinsic factors were examined. On each of four commercial piglet-producing farms, piglets from two or three batches of eight sows were studied. The piglets were included within 18 h after birth. Each piglet was individually scored four times. The score for the heels differentiated six (0–5) and for the coronary band and forelimb skin abrasion three stages (0–2). The body weight was measured two times. The effect of the floor was estimated by allocating the sows randomly to farrowing pens equipped with either soft rubber mats covered with litter or fully slatted plastic floors.

**Results:**

The final analysis comprised data from 1045 piglets. Foot lesions were not found at birth but started to develop on day 1. On day 5, heel bruising was found in 94%, main claw coronary band lesions in 49% and forelimb skin abrasion in 73% of the piglets. In a multifactorial logistic regression analysis, it was shown that a slatted plastic floor significantly increased the odds of heel bruising and coronary band lesions, while a rubber floor with litter increased the odds of forelimb skin abrasions.

**Conclusion:**

Foot and forelimb lesions in new-born piglets are mainly induced by the floor. The effect of slatted plastic floors on heel bruising showed an overwhelming OR of 52.89 (CI 26.29–106.43). Notably, coronary band lesions in young suckling piglets occur on slatted as well as non-slatted floors, indicating that the piglets incur these injuries not only from the wedging of their feet into the gaps between slats but also from contact with the floor while suckling. Based on these findings, preventive measures should be redirected to the improvement of the floor in the farrowing pen, particularly in the area under the sow’s udder.

**Supplementary Information:**

The online version contains supplementary material available at 10.1186/s40813-023-00351-9.

## Background

Foot lesions and forelimb skin abrasions in pigs presumably impair welfare and may reduce performance [1–4]. Whereas a noteworthy number of publications are focused on foot lesions in sows and fattening pigs, e.g., [5–13], only a limited number of publications address the same condition in suckling piglets. Foot lesions are a frequent occurrence in piglets, with a prevalence of up to 100% [14–20]. Forelimb skin abrasions have a reported prevalence of 70% or higher, while hairless patches in this area are seen in up to 90% of suckling piglets [2, 14, 21–23]. The prevalence and severity of foot and forelimb lesions vary by environment [3, 15]. Piglets with foot lesions or forelimb skin abrasions are less active than unaffected littermates in suckling, walking, playing and fighting [2, 16]. Additionally, lameness, associated with some claw lesions, indicates pain [24]. As abrasions, heel erosion and injury of the coronary band penetrate the epidermis, they provide an entry site for pathogens [16, 18].

The foot of a pig comprises the toe, sole, heel and wall [6]. The coronary band is the upper, almost circular limit of the wall and the zone of wall development [1]. The toe, sole and heel form the volar, weight-bearing surface [6]. At birth, the distal parts of the toe, sole, heel and wall are covered by the *Capsula ungulae decidua*, which is also referred to in some publications as the *eponychium* [25]. The *Capsula ungulae decidua* protects the amnion and uterus from injuries that can be induced by the activity of the foetus during gestation and birth [25]. Foot lesions in suckling piglets include bruising and erosion of the sole, heel, coronary band swelling and injuries [6, 15, 20]. When reviewing the literature, one should bear in mind that the term ‘sole’ is frequently applied to the entire volar surface when describing the locations of lesions, in most cases presumably lesions of the heel. Heel bruising consists of a haemorrhage into the corium [3, 15]. It is most prevalent in the first week of life, when the epidermis is very thin, and erosion arises when the heel epidermis is removed [15, 18]. The heels of new-born piglets are very vulnerable to bruising, as the high water content makes the horn extremely soft [2]. The negative association between heel bruising and age is probably because the epithelium of the heel is only 1–2 mm deep at birth and thickens with increasing age [18]. Alternatively, in an abrasive environment, heel bruising may be replaced by heel erosion [18]. Heel erosion is strongly associated with heel bruising [20].

Coronary band injuries have been considered in only a few publications [19, 20, 26]; as a result, little is known about these lesions [20]. Coronary band lesions are thought to arise when the claw is wedged into a gap between slats, leading to pressure and necrosis [1, 18].

Forelimb skin abrasions mainly occur in three sites: the carpus, metacarpus and digit [15]. Sometimes, the elbow or hock is also affected [16]. Carpal skin damage can be present from the day of birth and increases over the first week of life [14, 15, 27]. The mechanism of skin abrasion is mainly related to friction. Repeated rubbing of the limbs on the floor during suckling plays an important role in the distribution of abrasions on the front limbs. Additionally, the carpal area of the front limbs is especially susceptible to abrasions because of an increased concentration of force on this area when the carpus bears the body weight while piglets kneel during suckling [15]. There is a positive correlation between the total time spent suckling and the incidence of carpal skin abrasion between four and six days of age [2]. The presence of skin abrasions on the front limbs is significantly associated with the presence of heel bruising on the front feet [15]. The skin lesions are presumably also a result of contact with the floor, especially during suckling [15, 16]. Histopathology indicates that these skin abrasions appeared to move from superficial to deeper structures of the skin [2].

In the aforementioned publications, heel and coronary band lesions as well as carpal abrasions are interpreted as being caused by inadequacies of the floors frequently used to equip farrowing pens [1, 15, 18, 19]. Poor flooring is associated not only with foot and forelimb lesions but also with lesions at the teats, tail, elbows, hocks and face [14, 15, 23, 28]. The effect of various floor types on the development of foot lesions and forelimb abrasion has been confirmed in several studies [6, 15, 18–22, 24, 26–28]. Moreover, this association is emphasised by the low prevalence of heel bruising in suckling piglets on outdoor farms [18]. On these farms, piglets are born in huts set on soil with deep straw bedding [18], providing conditions very similar to the nests that are built by wild boar sows under natural conditions [29].

One working group is taking a different approach, hypothesising that lesions of the claws, teats, navel, and tail as well as at the tip or base of the ear are the outcome of SINS (swine inflammation and necrosis syndrome) [30]. Heel bruising and coronary band injuries—but not forelimb skin abrasions—are suspected to be indicative of SINS, which is assumed to induce foot lesions in nearly 100% of piglets [31]. SINS is described as an endogenous disease, and it is assumed that the pathogenesis is mainly determined by bacterial degradation products, such as lipopolysaccharides, and mycotoxins, particularly deoxynivalenol [32]. Suckling piglets are posited to be exposed in utero, leading to SINS-associated lesions already visible at birth, or through the sow’s milk [30, 33]. Flooring is assessed as a less prominent risk factor at this early age [31]. The diagnosis of SINS is based on clinical examination, as no laboratory diagnostic method has been validated yet.

The heel bruising and coronary band lesions described as characteristic effects of poor flooring [1, 2, 15, 18, 19, 24, 26, 28] are the same findings described to be associated with SINS [30]. Therefore, the objective of this study was to identify and quantify the effects of sex, litter size, body weight, average daily gain until day 5 and floor type on the occurrence of heel, coronary band and forelimb skin lesions in young suckling piglets. Learning more about the relevance of the various effects will enable veterinarians and farmers to focus on the main risk factor(s) when taking preventive measures against foot and forelimb lesions.

## Results

### Data description for foot lesions and forelimb skin abrasions

General scored health data in 1045 suckling piglets were described for the first 5 days of life.

#### Lesions on the right and left forefeet/hind feet and forelimbs in piglets at 5 days of age

On day 5, in more than 90% of the piglets, the scores for the heel, the forelimb and the coronary band of the dewclaw were the same on both sides (right/left). The coronary band of the main claw of the forelimb showed the same score bilaterally in 87% of the piglets, while the concordance of the hind legs was more than 90%. The findings in the right and left limbs are similar in the vast majority of piglets. For further evaluation, the decision was made to aggregate the findings from the right and left sides into to a single score, corresponding to the more severely lesioned side. On day 5, lesions of the heel and the coronary band of the main claw were found more frequently on the forelimbs than on the hind limbs (heel, *p* < 0.0001, coronary band *p* = 0.0227). The coronary bands of the dewclaws showed lesions in under 10% of the piglets, and no difference was found between the forelimbs and hind limbs.

#### Development of forefoot and forelimb lesions in piglets up to 5 days of age

Herein, it was focused on the development of lesions on the forelimbs and forefeet as exemplars of limb and foot lesions in general, as forelimb and hind limb lesions generally show no differences in development. All lesions seen on day 1 were seen exclusively after the detachment of the *capsula ungulae decidua*, while piglets that retained the *capsula ungulae decidua* showed no lesions. The detachment of the *capsula ungulae decidua*, examined in a small subset of piglets, occurred within 2.5 h after birth (Additional file 1, Removal of *capsula ungulae decidua* in a newborn piglet).

**Fig. 1:**
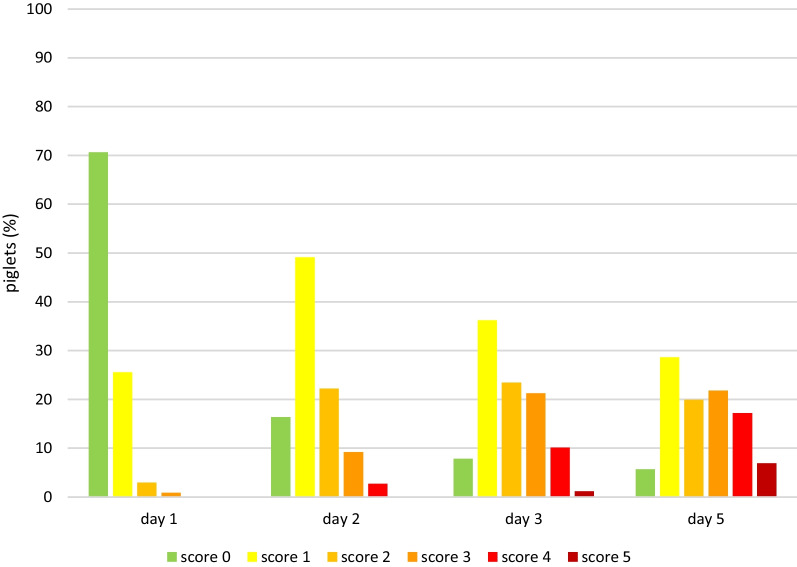
Development of forefoot heel bruising in suckling piglets (n = 1045) up to 5 days of age

**Fig. 2:**
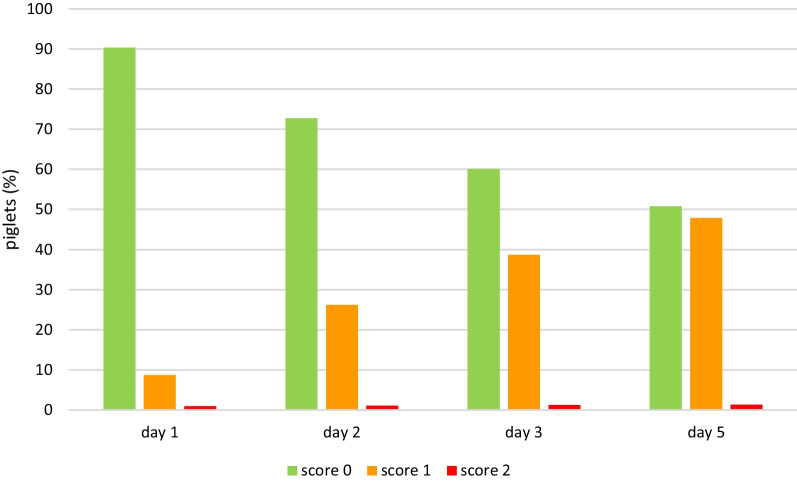
Development of forefoot main claw coronary band lesions in suckling piglets (n = 1045) up to 5 days of age

**Fig. 3:**
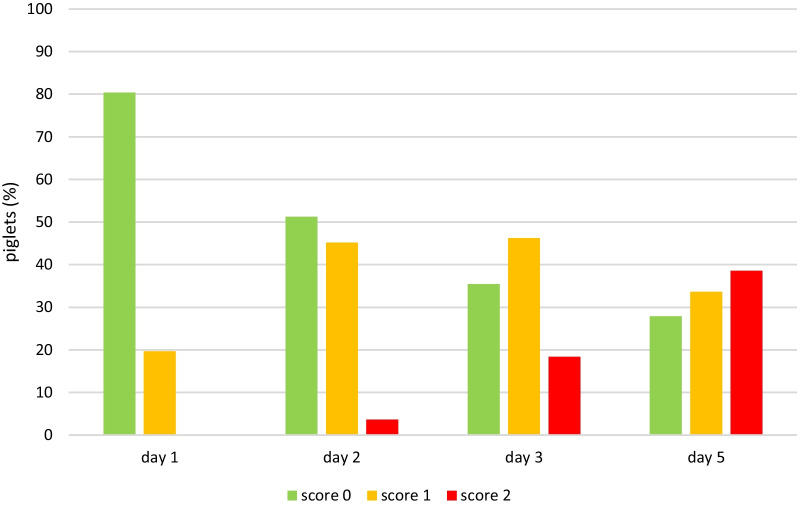
Development of forelimb skin abrasion in suckling piglets (n = 1045) up to 5 days of age

Lesions were already visible on day 1, when 29.4% of the piglets showed mild heel bruising. By day 5, heel bruising had developed in 94% of the piglets, showing increasing severity (Fig. 1). Heel bruising developed rapidly, showing a statistically significant increase between days 1 and 2, between days 2 and 3 and between days 3 and 5 (*p* < 0.0001). On day 5, the odds of heel bruising were 61.4 times higher than on day 1 (CI 44.67–84.49). Focal coronary band lesions of the main claw were seen in 8.7% of the piglets on day 1, while 47.9% were affected on day 5. Diffuse coronary band lesions were seen on day 5 in 1.3% of the piglets, all belonging to one litter (Fig. 2). Due to the low number of affected piglets, diffuse coronary band lesions were added to score 1 for further data analysis. The development of main claw coronary band lesions showed a statistically significant increase between days 1 and 2 (*p* < 0.0001) and between days 2 and 3 (*p* = 0.0224). On day 5, the odds of coronary band lesions were significantly higher than on day 1 (OR 10.03, CI 7.86–12.80). Superficial forelimb skin abrasions were found in 19.6% of the piglets on day 1 (Fig. 3). By day 5, 34% of the piglets showed superficial lesions (score 1), while 39% developed lesions with secretions or scabs (score 2). Forelimb skin abrasions increased significantly between days 3 and 5 (*p* < 0.0001). On day 5, the odds of forelimb skin abrasions were 13.6 times higher than on day 1 (CI 10.96–16.98). The coronary bands of the dewclaws (fore and hind feet) showed focal lesions in approximately 8% of the piglets on day 5. Spot bleeding into the main claw was a rare finding, seen in 19 piglets (1.8%) belonging to 9 litters.

#### Similarities between foot and forelimb lesions in piglets at 5 days of age

The foot and forelimb findings on day 5 were tested for similarities. Forefoot and hind foot lesions showed moderate similarity (kappa measure 0.49), while most of the other lesions showed very low similarity, with kappa measures close to zero and asymmetry confirmed by the McNemar test (Table 1).

**Table 1:** Similarities between foot and forelimb lesions analysed with Cohen's κ (upper right triangle) and *p* value of McNemar’s test (lower left triangle; statistically significant asymmetry is marked italic)

	HB-FL	CBM-FL	CBD-FL	SA-FL	HB-HL	CBM-HL	CBD-HL
HB-FL	1	0.0755	0.0032	0.1108	0.4874	0.0741	0.0076
CBM-FL	* < 0.0001*	1	0.0125	0.0917	0.0198	0.4672	0.0166
CBD-FL	* < 0.0001*	* < 0.0001*	1	0.0258	0.0060	0.0183	0.0392
SA-FL	* < 0.0001*	* < 0.0001*	* < 0.0001*	1	0.0152	-0.0164	0.0167
HB-HL	* < 0.0001*	* < 0.0001*	* < 0.0001*	* < 0.0001*	1	0.0181	0.0061
CBM-HL	* < 0.0001*	*0.0227*	* < 0.0001*	* < 0.0001*	* < 0.0001*	1	0.0181
CBD-HL	* < 0.0001*	* < 0.0001*	0.5020	* < 0.0001*	* < 0.0001*	* < 0.0001*	1

*HB-FL* heel bruising, forefoot, *CBM-FL* coronary band lesion, main claw, forefoot, *CBD-FL* coronary band lesion, dewclaw, forelimb, *SA-FL* skin abrasion, forelimb, *HB-HL* heel bruising, hind foot, *CBM-HL* coronary band lesion, main claw, hind foot, *CBD-HL* coronary band lesion, dewclaw, hind limb

### Factors related to forefoot and forelimb lesions in piglets at 5 days of age

The association of heel bruising, main claw coronary band lesions and forelimb skin abrasions with extrinsic factors was modelled in a logistic regression analysis (heel bruising, Table 2; coronary band lesions, Table 3; forelimb skin abrasion, Table 4). The comparison of the ORs from the univariable model with those from the multivariable model showed that any confounding of those factors evaluated to have a statistically significant impact was unlikely.

**Table 2:** Logistic regression model for intrinsic and extrinsic variables related to heel bruising in piglets at 5 days of age

Risk categories	Heel bruising				Univariable model			Multivariable model		
	Score 0/1/2		Score 3/4/5		OR	95% CI		OR	95% CI	
	n	%	n	%		Lower bound	Upper bound		Lower bound	Upper bound
Piglet's sex					*p* = 0.2030			*p* = 0.3527		
Female	270	52.63	243	47.37	1	×	×	1	×	×
Male	270	53.68	233	46.32	1.282	0.874	1.879	1.211	0.809	1.812
Litter size					*p* = 0.8651			*p* = 0.9083		
< 16	283	53.00	251	47.00	1	×	×	1	×	×
≥ 16	283	54.32	238	45.68	0.909	0.30	2.730	0.960	0.480	1.920
Floor					*p* < 0.0001			*p* < 0.0001		
RML*	443	82.80	92	17.20	1	×	×	1	×	×
PSL*	123	24.12	387	75.88	40.590	21.133	77.961	52.891	26.286	106.425
Body weight day 1 (kg)					*p* = 0.0003			*p* = 0.0006		
≤ 1.20	185	58.18	133	41.82	1	×	×	1	×	×
1.21–1.50	161	51.94	149	48.06	2.543	1.510	4.281	2.541	1.472	4.385
≥ 1.51	151	49.51	154	50.49	3.085	1.677	5.676	3.071	1.626	5.801
Average daily gain until day 5 (kg)					*p* = 0.0454			*p* = 0.7105		
≤ 0.110	157	53.04	139	46.96	1	×	×	1	×	×
0.111–0.0160	173	56.17	135	43,83	1.136	0.694	1.859	1.092	0.661	1.802
≥ 0.161	158	51.47	149	48.53	1.911	1.108	3.294	1.275	0.713	2.281

*RML soft rubber with litter (wood shaves), *PSL* plastic slats

**Table 3:** Logistic regression model for intrinsic and extrinsic variables related to main claw coronary band lesions in piglets at 5 days of age

Risk categories	Heel bruising				Univariable model			Multivariable model		
	Score 0/1/2		Score 3/4/5		OR	95% CI		OR	95% CI	
	n	%	n	%		Lower bound	Upper bound		Lower bound	Upper bound
Piglet's sex					*p* = 0.1409			*p* = 0.1143		
Female	279	54.39	234	45.61	1	×	×	1	×	×
Male	241	47.91	262	52.09	1.263	0.926	1.722	1.290	0.940	1.770
Litter size					*p* = 0.0179			*p* = 0.0107		
< 16	303	57.82	221	42.18	1	×	×	1	×	×
≥ 16	228	43.76	293	56.24	2.246	1.150	4.385	2.166	1.197	3.918
Floor					*p* < 0.0001			*p* < 0.0001		
RML*	344	64.30	191	35.70	1	×	×	1	×	×
PSL*	187	36.67	323	63.33	3.710	2.093	6.575	3.995	2.296	6.951
Body weight day 1 (kg)					*p* = 0.1616			*p* = 0.1216		
≤ 1.20	191	60.06	127	39.94	1	×	×	1	×	×
1.21–1.50	148	47.74	162	52.26	1.486	0.989	2.232	1.546	1.012	2.363
≥ 1.51	145	47.54	160	52.46	1.288	0.811	2.045	1.416	0.872	2.301
Average daily gain until day 5 (kg)					*p* = 0.4048			*p* = 0.2854		
≤ 0.110	159	53.72	137	46.28	1	×	×	1	×	×
0.111– 0.0160	161	52.27	147	47.73	1.289	0.857	1.937	1.288	0.854	1.942
≥ 0.161	154	50.16	153	49.84	1.042	0.676	1.609	0.951	0.602	1.503

*RML soft rubber with litter (wood shaves), *PSL* plastic slats

**Table 4:** Logistic regression model for intrinsic and extrinsic variables related to forelimb skin abrasion in piglets at 5 days of age

Risk categories	Heel bruising				Univariable model			Multivariable model		
	Score 0/1/2		Score 3/4/5		OR	95% CI		OR	95% CI	
	n	%	n	%		Lower bound	Upper bound		Lower bound	Upper bound
Piglet's sex					*p* = 0.6232			*p* = 0.6742		
Female	141	27.49	372	72.51	1	×	×	1	×	×
Male	148	29.42	355	70.58	0.923	0.671	1.270	0.933	0.647	1.291
Litter size					*p* = 0.1080			*p* = 0.1726		
< 16	170	32.44	121	23,22	1	×	×	1	×	×
≥ 16	354	67.56	400	76.78	1.604	0.901	2.856	1.473	0.844	2.572
Floor					*p* = 0.0246			*p* = 0.0176		
RML*	121	22.62	414	77.38	1	×	×	1	×	×
PSL*	170	33.33	340	66.67	0.0527	0.302	0.921	0.514	0.297	0.890
Body weight day 1 (kg)					*p* = 0.1711			*p* = 0.1861		
≤ 1.20	114	35.85	204	64.15	1	×	×	1	×	×
1.21–1.50	82	26.45	228	73.55	1.253	0.822	1.907	1.427	0.917	2.221
≥ 1.51	89	29.18	216	70.82	0.820	0.516	1.304	1.016	0.619	1.669
Average daily gain until day 5 (kg)					*p* = 0.1101			*p* = 0.0742		
≤ 0.110	78	26.35	218	73.65	1	×	×	1	×	×
0.111–0.0160	91	29.55	217	70.45	0.843	0.550	1.291	0.797	0.518	1.225
≥ 0.161	112	36.48	195	63.52	0.628	0.403	0.979	0.578	0.359	0.929

*RML soft rubber with litter (wood shaves), *PSL* plastic slats

#### Sex of the piglets

An effect of sex was not found, as forefoot and forelimb lesions were seen in male and female piglets with similar frequencies.

#### Litter size at birth

The number of live-born piglets (day 1) had no influence on the occurrence of heel bruising or forelimb skin abrasions on day 5. However, increasingly severe main claw coronary band lesions were found in piglets belonging to litters with 16 or more live-born piglets (OR 2.16, CI 1.20–3.92; *p* = 0.0107).

#### Floor

Heel bruising and main claw coronary band lesions were seen more frequently and at greater degrees severity in piglets kept on slatted plastic floors than in piglets kept on rubber mats with litter. The effect of slatted plastic floors on heel bruising was confirmed by an OR of 52.89 (CI 26.29–106.43; *p* < 0.0001). In contrast, the slatted plastic floors had a ‘protective’ effect against forelimb skin abrasion (OR 0.51, CI 0.30–0.89; *p* = 0.0176), although this lesion type was still frequently seen in piglets kept on plastic slats as well as on rubber mats with litter.

#### Body weight at birth

Piglets that developed heel bruising scores ≥ 3 by 5 days of age were heavier at birth than piglets with heel bruising scores ≤ 2 (OR 3.07, CI 1.63–5.80). An effect of body weight at birth was not shown for the development of main claw coronary band lesions or forefoot skin abrasion.

#### Average daily gain from birth to day 5

The average daily gain calculated for the period from birth to day 5 showed no effect on heel bruising, main claw coronary band lesions or forefoot skin lesions.

## Discussion

In several studies, poor flooring was identified as the main risk factor for foot lesions in new-born piglets; recently, however, the same clinical findings were suspected to be typical of SINS. The hypothesis of poor flooring follows the assumption that heel bruising, coronary band lesions and forelimb skin abrasions develop when feet and limbs come in contact with a hard or rough floor [1, 15, 18, 19, 21, 22, 26, 27]. Consequently, the lesions are restricted to locations that touch the floor while the piglet is moving or suckling [14, 15, 23, 28]. The frequency and severity of the lesions are influenced by the type of floor and presumably by additional factors such the activity [2] or body weight of the individual or the size of the litter. Furthermore, it was hypothesised that these lesions start to develop just after birth and evolve from ‘top to bottom’ [2]. The other hypothesis is based on the assumption that a condition known as SINS, which is suspected to be an endogenous disease, is the main risk factor for heel bruising and coronary band lesions [30, 31]. The endogenous pathogenesis of the disease leads to the expectation that the lesions would develop from ‘bottom to top’ and would occur even in locations that did not necessarily touch the floor. Moreover, it was expected that the intrauterine development of SINS [31] would result in lesions visible at birth.

### Frequencies and development of lesions over time

Foot and forelimb lesions were already visible on the day of birth—but not at birth—and increased in severity and frequency until Day 5. This observation corresponds well to the timeline and frequencies reported in other studies [2, 14–22, 28]. In new-born piglets that still had an intact *capsula ungulae decidua*, no lesions were found. All piglets showing early stages of the lesions had mostly or totally lost their *capsula ungulae decidua* by movement. The follow-up of a small subsample of piglets showed that the *capsula ungulae decidua* was fully detached within 1.5 to 2.5 h after birth. Consequently, the scoring of piglets within 2 h after birth, intended to examine SINS in new-born piglets [34], cannot exclude lesions induced by the environment. The intrauterine development of foot lesions, which is suspected to be a sign of SINS, needs to be demonstrated in piglets that still have an intact *capsula ungulae decidua*. Obviously, this can be guaranteed only when the piglets are examined before touching the floor.

### Distribution of lesions on forelimbs and hind limbs

In most piglets, lesions were found bilaterally, while lesions restricted to one limb were approximately equally distributed on the right and left, as already described by [15]. On day 1, the forefeet and hind feet were equally affected, but on Day 5, lesions were more frequently found on the forefeet than on the hind feet. This observation was also confirmed by [20], but other studies showed no difference [15] or more lesions on the hind feet [19, 22]. The increased prevalence of heel bruising on the forefoot might be influenced by the factor of body weight, which is mainly borne by the forefeet [35].

### Similarities between foot and forelimb lesions

Heel bruising on the forelimb appeared similar to heel bruising on the hind limb, with a kappa measure of 0.49, indicating that the lesions appear at the same time and therefore are likely induced by the same factor. For all other lesions, the kappa measure reflected a low to very low positive association, and asymmetry was confirmed by the McNemar test. The results indicate that the lesions do not necessarily appear at the same time and that their development might be influenced by lesions that developed earlier. Heel bruising, for instance, might cause pain that would result in longer kneeling on the carpus, enhancing the risk of skin lesions. The time delay in the appearance of the lesions might also (or even exclusively) be an effect of the differences in the mechanical burden. While the heels are burdened by all locomotion and suckling, the other locations are burdened almost exclusively during suckling. Studies linking forefoot heel bruising and coronary band lesions with forelimb skin abrasion have already been published [15, 20], while other associations or similarities do not appear to have been analysed before. Foot lesions and dewclaw coronary band lesions of the forelimb are consistent with the posture that piglets assume when suckling. Piglets usually stand when suckling on teats in the upper row, but when they suckle from teats in the lower row, they need to ‘kneel’ on their forelimbs and position the hind limbs either close to the belly or extended behind the body (Additional file 2, Variation in suckling-postures of new-born piglets lead to contact of different localisations of fore and hint limbs with the floor). In this posture, the main claw coronary bands of the forelimb and hind limb, the carpus and metacarpus of the forelimb and sometimes the lateral part of the dewclaw coronary band of the hind limb are necessarily in close contact with the floor. The activity of the piglet, which is necessary to initiate milk letdown, very likely enhances the risk of abrasion and bruising of the aforementioned tissues. In contrast to the aforementioned locations, the dewclaw coronary bands of the forelimbs are protected from direct floor contact by the flexed carpal joints when the piglet is suckling. The forelimb dewclaw coronary bands usually come into contact with the floor when the piglet is resting in sternal recumbency with outstretched limbs. Heel bruising and coronary band lesions are signs associated with SINS, which is suspected to affect the circulation in areas fed by terminal vessels (claws, tail, ear tips, and teats), resulting in inflammation and necrosis [30]. However, the hypothesis forming the basis of SINS does not answer the question of why heel bruising and coronary band lesions are associated with carpal and metacarpal skin abrasions, as these areas are clearly not fed only by terminal vessels.

### Factors related to forefoot and forelimb lesions in piglets at 5 days of age

The association of heel bruising, main claw coronary band lesions and forelimb skin abrasions with intrinsic and extrinsic factors was tested by logistic regression analyses on binary outcomes using the GLIMMIX procedure. As expected, no effect of sex on the occurrence of heel bruising, main claw coronary band lesions or forefoot skin abrasion was observed. An effect of sex on foot lesions was also not reported in any other study. Piglets born in litters of 16 or more live-born piglets developed more frequent and more severe main claw coronary band lesions than piglets from smaller litters. This effect might be a result of enhanced activity in larger litters. Activity, fighting and the development of social relations in new-born piglets generally start just after birth [21, 36] and are influenced by the availability of functional teats [16, 28], which decreases with increasing litter size.

First, the effect of the floor on foot and forelimb skin lesions requires a critical evaluation of the two flooring types used in this study. Floors in farrowing pens present a dilemma because the needs of the sow differ from those of the piglets in a number of ways [19, 28]. Piglets need floors with low abrasiveness, while the sow needs abrasive and, to a certain degree, rough flooring to prevent claw overgrowth and slipping [28]. Floors for farrowing pens, primarily used in conventional piglet-producing farms, are fully or at least partly slatted and are made of concrete, expanded metal, plastified metal, plastic or a combination of these materials. Although significant flooring-dependent differences in the frequency and severity of foot and forelimb lesions have been identified, no single floor type is ideal for piglet foot health [6]. Based on these results, foot and forelimb skin lesions, which typically develop on slatted plastic floors and even more on concrete floors, were also expected in this study. To compare the effect of two different flooring types, the decision was made to upgrade the floor for 50% of the sows and their litters by adding bedding, while 50% were housed on a slatted plastic floor. The presence of straw bedding on concrete or rubber-covered floors is associated with reduced incidence rates of heel and coronary band lesions [22, 23, 27, 28, 37] but cannot reduce the lesion rate to nearly zero, as is possible with straw bedding on soil [18]. In the rubber mat/litter group of this study, wood shavings were used for bedding, as the surface is rougher compared to straw, which is known to be scraped away easily by the piglets, especially during suckling [27, 28]. However, wood shavings and sawdust also failed to completely prevent damage when used to cover concrete floors [22, 23, 28]. Peat, also known to be very effective in preventing carpal abrasion and heel lesions [19, 28], is available only for gardening in Germany, and its origin is not specified. Consequently, this option was refused due to hygienic concerns. To inhibit the wood shavings from dropping through the slats and blocking the liquid manure system, it was decided to cover the plastic slats in the entire pens with soft rubber mats. Only behind the sow was a small part of the slatted floor kept open to facilitate the drainage of the sow’s urine. Unexpectedly, the large amount of wood shavings was also scraped away by most of the litters during suckling or by the sow. Therefore, most piglets in the rubber mat/litter group came directly in contact with the rubber mat with various parts of the body when suckling. In the creep area, the wood shavings were not scraped away, suggesting that the lesions developed only during suckling.

Heel bruising and main claw coronary band lesions were seen with greater frequency and severity in piglets that were kept on slatted plastic floors than in those that were kept on rubber mats. The overwhelming effect of the slatted plastic floors on the development of heel bruising was confirmed by an OR of 52.89 (CI 26.29–106.43). In contrast, the slatted plastic floors had a slight ‘protective’ effect against forelimb skin abrasion (OR 0.51, CI 0.30–0.89), as piglets kept on plastic slats showed fewer forelimb skin lesions (66.67%) than those kept on rubber mats with litter (77.38%). The results clearly confirm the pronounced effect of the floor on the development of foot lesions in piglets up to 5 days of age. None of the other tested factors showed a comparable effect. Rubber mats covered with wood shavings prevent heel bruising and coronary band lesions in new-born piglets. Although the preventive effect was obvious, coronary band lesions were still seen in 35% of the piglets housed on rubber mats/litter. This finding requires a critical evaluation of the hitherto favoured hypothesis regarding the pathogenesis of coronary band lesions, in which they result from the wedging of the claws into the gaps between slats, leading to pressure and necrosis [1, 18]. The appearance of coronary band lesions in piglets housed on non-slatted rubber mats indicates another pathogenesis in addition to that already known. The aforementioned ‘kneeling’ posture of piglets suckling on teats in the lower row inevitably brings the coronary band into close contact with the floor (Additional file 2c). This contact is intensified by the activity of the piglet that is necessary to initiate milk letdown. Foot and forelimb skin lesions induced by the floor during suckling activity should redirect the focus to the improvement of the floor under the udder. As piglets show the highest activity in different postures in this area of the pen, the development of floors meeting the needs of new-born piglets would raise the standard of welfare. Although rubber mats with litter protect piglets from heel bruising and coronary band lesions to a certain degree, this floor type was found to be far from being perfect for young suckling piglets, as forelimb skin abrasions appeared more frequently and wounds were larger in diameter and deeper. This effect was previously described by [21]. Although the abrasiveness of rubber is low [27], the characteristic friction of soft rubber [38] likely induces distinct shear forces. It can be concluded that the floors used in this study are different in hardness (plastic slats) and shear forces (rubber mats), but both have the potential to damage the feet and forelimb skin of suckling piglets. With the exception of one litter, coronary band lesions of the main claws appeared focally on the anterior or lateral aspect. The interdigital part of each claw’s coronary band, which is protected from contact with the floor, was unaffected. These findings support the hypothesis that contact with the floor induced the lesions, while signs indicating a systemic, endogenous disease were not observed. In only one out of the 88 litters, diffuse lesions extending to the interdigital part of the main claw coronary band were found, while the dewclaw coronary band was unaffected. The reason for this clearly differing finding has not been identified. Lesions of the dewclaws, seen in approximately 8% of the piglets, were located only on the plantar side, also suggesting that they were induced by contact with the floor.

In agreement with a previous report [2], birth weight has an effect on the development of heel bruising. Lightweight piglets showed significantly less frequent and less severe heel bruising than piglets with higher birth weight. Higher birth weight might influence heel bruising by increasing the pressure on the weight-bearing surface of the foot. Moreover, the viability of piglets is strongly associated with birth weight [39]. It is very likely that more active piglets have a higher risk of heel bruising. An effect of heel bruising on average daily gain up to day 5 was not confirmed but cannot be excluded by this study. At first glance, the results appear to show that heel bruising does not affect performance and that welfare is obviously not impaired. This interpretation is likely premature, as average daily gain is not a sensitive parameter for the evaluation of welfare in pigs. Moreover, the observation period (until day 5) was likely too short to evaluate the effect of severe lesions just appearing on day 3. Consequently, this result does not conclusively refute studies [2, 16] confirming a negative effect of foot lesions and forelimb skin abrasion on the behaviour of affected piglets.

In the models, the variables of farm, batch and sow were included as random factors to take the different frequencies of observed lesions per farm and the different ages of the sows into account. Farm and batch have been identified as risk factors for many infectious and non-infectious diseases and injuries [40, 41]. In general, farm, batch and sow summarise the effect of a wide variety of factors—e.g., the health status, particularly PPDS, of the herd and individuals, housing, management, hygiene, the human-pig relationship, and nutrition—that cannot be further discriminated in this study.

In addition to the factors considered as fixed or random factors in this study, others that might have contributed to the development of heel bruising, coronary band lesions and/or forelimb skin abrasion should also be discussed. The association between claw lesions and flooring in young suckling piglets is temporally consistent with insufficient coordination just after birth [19] and early activity in new-born piglets [42, 43]. In various studies on the agonistic behaviour of piglets and the development of social relations, piglets were observed to fight frequently during the first few hours after birth [36]. This behaviour suggests that lesions may occur in the early life of piglets [21]. New-born piglets start suckling just after birth, at which time the suckling behaviour appears disorderly and is clearly different from the highly organised pattern apparent in the next few days. During the first hours after birth, suckling bouts are frequent, and the piglets use different teats. Early suckling is already characterised by fights to gain access to a functional teat [42]. The suckling and agonistic behaviour in the time just after birth clearly show that lesions caused by movement are very likely and cannot be excluded, as suggested by [31]. The effect of disinfectant powder (e.g., Stalosan®) extensively used on farms in daily practice to keep farrowing pens dry should be considered in further studies. The low pH (4.5) of Stalosan® (www.soan.at, Stalosan F Sicherheitsdatenblatt (EC) 1907/2006) might irritate the heel horn, which is very vulnerable due to its high water content in new-born piglets. Moreover, other factors not confirmed in this study have also been associated with the occurrence of claw lesions, including genetic predisposition [13, 44] and lack of biotin in the diet [45]. The influence of genetics might, at least to a certain degree, be associated with pigmentation, as the volar surface of pigmented breeds is tougher and thicker (melanin increases the strength of keratinised tissue) and therefore provides more protection against heel/sole erosion [6]. SINS is also suspected to be influenced by genetics, as the offspring of Duroc boars showed lower lesion scores than those of Pietrain boars [46]; however, the role of pigmentation [6], typical of the integument of Duroc pigs, was not discussed.

At present, the diagnosis of SINS is made exclusively based on clinical signs, as a validated laboratory method appropriate for the detection of the endogenously induced inflammation that is suspected to induce SINS is not available. The heel and coronary band lesions found in this study exactly match those that have been associated with SINS [31, 33, 47]. Nonetheless, the results of this study clearly show that foot lesions in new-born piglets are induced and modulated by the floor as a main driver.

## Conclusion

Heel bruising, focal coronary band lesions of the main claws and dewclaws and forefoot skin abrasions are frequently seen in young suckling piglets. Early signs of the lesions appeared within a short time after birth, but there was no evidence that the lesions had developed in utero. Lesions were restricted to localisations that directly contacted the floor during suckling. This is noteworthy particularly for main claw coronary band lesions, which clearly evolved not only on slatted floors, with gaps in which the claws could become wedged, but also on flat floors. The distribution and characteristics of the lesions suggest that the floor plays a prominent role in development. Efforts to optimise floors in farrowing pens, especially in the area where the sow lies when suckling the litter, are urgently required to prevent or reduce these highly prevalent lesions. The suggestion that foot lesions are mainly induced by SINS and that this can be easily diagnosed in a clinical examination is not supported by this study. As heel bruising and coronary band lesions, the most prevalent findings suspected to be typical of SINS, were found in this study to be closely associated with damage induced by the floor, a validated laboratory diagnostic is inevitably necessary to confirm the presence of SINS.

## Methods

### Farm selection

The study was carried out as a longitudinal trial on four commercial piglet-producing farms [1–4] in northwestern Germany (Table 5). As usual in commercial piglet production in Germany, all sows were crated permanently during the observation period. The farms were selected by convenience sampling based on the following criteria: (1) farm location within 100 km from the Field Station for Epidemiology of the University of Veterinary Medicine Hannover; (2) piglet production based on batch farrowing with at least eight farrowing pens per unit; and (3) willingness of the farmer to participate in the study. In the study herds, piglets routinely undergo the following routine procedures during day 1: tail docking, teeth grinding and iron substitution by injection. Antimicrobials were routinely applied in farm 3 and 4 (day 1), and anticoccidials in farm 2 (day 1), farm 3 and 4 (day 3).

**Table 5:** Number of animals and housing conditions on participating farms

	Farm 1	Farm 2	Farm 3	Farm 4
Genetic sows	DanBred	DanBred and Topigs TN70	Topigs TN70	Topigs TN 70
No. of sows in herd	270	450	120	430
No. of sows and litters in study	24	24	16	24
Average litter size (live-born piglets)	15.4	15.1	14.6	16.2
No. of piglets entering study	364	358	239	386
No. of piglets incl. in data analysis	299	294	161	291
No. of piglets housed on RML^+^ PSL^+^	147 152	150 144	85 76	153 138
Size of farrowing pen Type of PSL Slat width	2.6 × 1.8 m A* 10 mm	2.1 × 2.5 m B* 10 mm	2.3 × 2.1 m C* 15 mm	2.5 × 1.6 m C* 15 mm
Width of slat voids	10 mm	10 mm	10 mm	10 mm
Disinfectant powder, dispersed on slatted floor in PSL before farrowing	Ökosan^®^	Stalosan^®^	Stalosan^®^/Lactisec^®^	None

^+^*RML* soft rubber with litter (wood shaving), *PSL* plastic slats; *A, slatted plastic floor; plain plastic floor in the area where the sow lay; *B, slatted plastic floor; slatted cast iron in the area where the sow lay; *C, slatted plastic floor with slightly elevated bars on the slats; plain plastic floor in the area where the sow lay; Ökosan^®^ (GFR mbH, 970,080 Würzburg, Germany), Stalosan^®^ (Stormøllen A/S, 4682 Tureby, Denmark), Lactisec^®^ (Universal Kraftfutterwerk Kehl, 77,694 Kehl, Germany)

### Sample size

On each farm, two or three consecutive batches of eight sows each were investigated (Table 5). On farm 1, three batches were studied, but in the first batch, the body weight data were not collected. On farm 3, the study was cancelled after two batches due to hygienic concerns expressed by the farmer after the first outbreak of African swine fever in the wild boar population in Germany in September 2020. In total, 1347 piglets from 88 litters were included in the study on day 1. The final analysis comprised data from 1045 piglets, while 302 piglets were excluded because compete data were not available due to cross-fostering (to litters not involved in the study) or death. One sow (farm 3) was excluded as a caesarean section was necessary and piglet mortality was enhanced due to this incident. This sow was replaced by another one from the same batch.

### Clinical findings and body weight

**Fig. 4:**
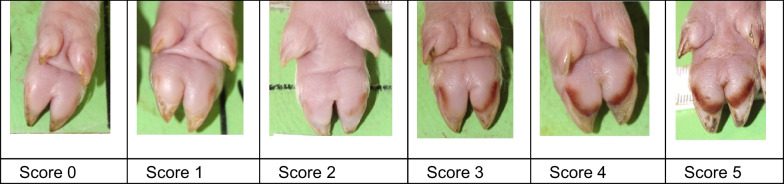
Heel bruising score

**Fig. 5:**
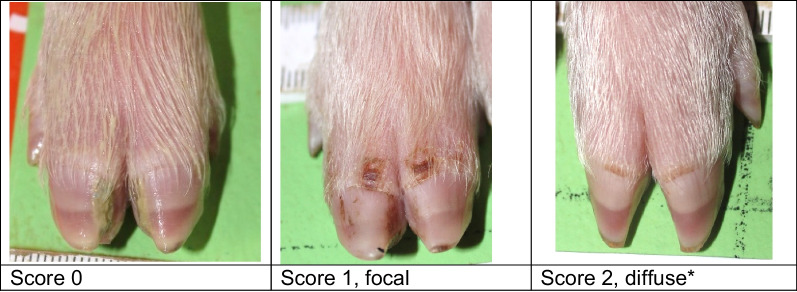
Coronary band lesion score. * Score 2 was seen in piglets from only one litter

**Fig. 6:**
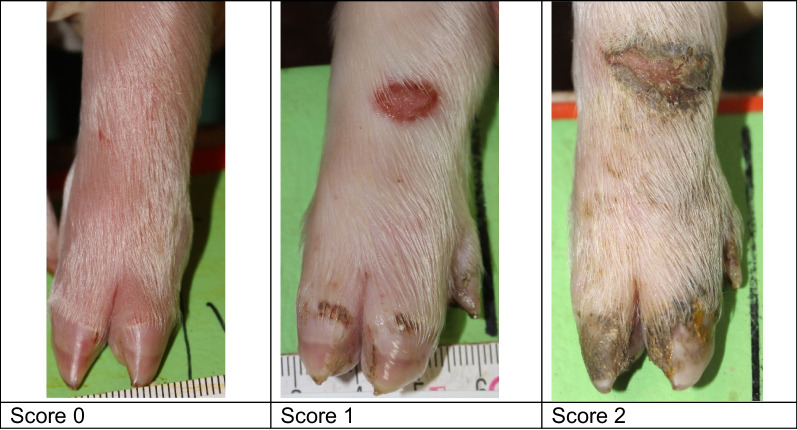
Forelimb skin abrasion score

All piglets in each eligible litter were included in the study within 18 h after birth (day 1). Photographs were taken of all four feet (top and bottom view) and the forelimb skin of each piglet on days 1, 2, 3 and 5. Heel bruising, coronary band lesions and forelimb skin abrasions were scored (Table 6, Figs. 4, 5, 6). Other foot lesions, such as heel erosion, wall cracks and wall bleeding, were also recorded but were not scored due to their rare occurrence. Sole bruising was not recorded, as the very small tip of the volar surface shows a slight convexity, which, in new-born piglets is frequently filled with a mixture of faeces, navel blood, amniotic fluid, urine and litter, making the examination of large sample sizes impossible. Hock and elbow abrasions were not recorded systematically. The scoring was performed, based on the photographs, by two trained veterinarians (MH and EgB), each scoring the entire dataset independently. In case of differences, the findings were discussed and the score was re-evaluated by both veterinarians.

**Table 6:** Lesion score

Score	Heel bruising	Coronary band injury*	Forelimb skin abrasion**
0	No lesion	No lesion	No lesion
1	One punctate lesion on the volar surface of the medial and/or lateral claw	Lesion, focal	Skin abrasion without serous secretion
2	Two or more punctate lesions on the volar surface of the medial and/or lateral claw	Lesion, diffuse	Skin abrasion with serous secretion or scab
3	Lesions converging in a semilunar shape on the volar surface of the medial or lateral claw	−	−
4	Lesions converging in a semilunar shape on the volar surfaces of the medial and lateral claws	−	−
5	Lesions beyond a semilunar shape on the volar surface of the medial or lateral claw	−	−

*The coronary bands of the main claws and dewclaws were scored separately

**Forelimb skin abrasions comprise lesions on the carpus and/or metacarpus

On a small subset of piglets (n = 12, one litter) included immediately after birth, the state of the *capsula ungulae decidua* was monitored until total detachment.

Body weight was measured on days 1 and 5 with a paediatric scale (AE ADAM, model MTB 20, weight scale up to 20 kg with 0.005 kg steps; www.adamequipment.com). Average daily gain was calculated from these data. Furthermore, the sex of the piglets and the number of litters the sow had farrowed (‘sow age’) were documented.

Individual marking was performed by writing numbers with a permanent marker on the backs of the piglets, as additional ear tagging would not have been in accordance with the German animal welfare act.

### Farrowing pen, floor type and equipment

For each farrowing batch studied, floor types were randomly assigned to eight pens. Four farrowing pens (RML) were fully equipped with soft rubber mats (BELMONDO classic, Gummiwerk Kraiburg Elastik GmbH Co.KG, 84,529 Tittmoning, Germany), leaving only a 5 cm × 1.5 cm space behind the sow to facilitate the drainage of the sow’s urine. The rubber floor was covered with a 15 cm layer of wood shavings (Goldspan®, Goldspan GmbH & Co. KG, 49,424 Goldenstedt, Germany) two days before farrowing was expected. Additional wood shavings (volume: 10 L) were provided daily until the end of the monitoring period, when the piglets were 5 days old. The other four farrowing pens remained unchanged. On all farms, the farrowing pens were equipped with a slatted plastic floor (PSL) and a solid plastic floor with integrated heating in the creep area (for details, see Table 5).

For additional heating, an infrared lamp was installed over the creep area in the pens of both study groups. Nothing was changed in the management of the sows from farrowing until the end of the monitoring period.

The 8 sows were randomly allocated to the pens 10 to 7 days before the expected date of farrowing.

### Data analysis

Data were organised in Microsoft Excel and transferred into the statistical analysis program SAS (version 9.4 TS level 1M5, SAS Institute Inc., Cary, NC, USA) for further statistical evaluation. For data description, foot lesions and forelimb skin abrasions were scored in up to six categories from 0 (no findings) to 6 (severe findings), as outlined in Table 6.

For statistical testing and modelling, binary outcomes were used. As an example, for heel bruising, the score was reduced to two categories by summarising scores as 0, 1, and 2 vs. 3, 4 and 5, as the modelling requires a minimum number of cases per cell, and only 7 piglets housed on rubber mats/litter showed score 4 and none showed score 5 for heel bruising. Similar categorisation was applied for sow age (i.e. number of litters) and litter size (< 16 vs. ≥ 16). Body weight on day 1 (≤ 1.120, 1.121 to 1.50, ≥ 1.51 kg) and average daily gain until day 5 (≤ 0.110, 0.111 to 0.160,  ≥ 0.160 kg). To investigate and test the similarity of outcomes, 2 × 2 tables were studied via Cohen's κ and the McNemar test. The association of heel bruising, main claw coronary band lesions and forelimb skin abrasions with extrinsic factors was modelled in a logistic regression analysis on binary outcome using the GLIMMIX procedure. For these models, farm, batch and sow were included as random factors to take the different frequencies of observed lesions per farm and the different ages of the sows into account. Other intrinsic and extrinsic factors (piglet’s sex, litter size, floor, body weight day 1, average daily gain until day 5) were included as fixed effects. P values less than 5% were considered statistically significant with no adjustment for multiple comparisons due to the exploratory nature of the study design.

### Supplementary Information

**Additional file 1:** Removal of* capsula ungulae decidua* in a newborn piglet.

**Additional file 2:** Variation in suckling-postures of new-born piglets lead to contact of different localisations of fore and hint limbs with the floor.

## Data Availability

Please contact the corresponding author for data requests.

## Abbreviations

PSL: Plastic slats
RML: Rubber mat plus litter
SINS: Swine inflammation and necrosis syndrome

## References

[1] Geyer H, Troxler J (1988). Klauenerkrankungen als Folge von Stallbodenmängeln. Tierärztl Prax.

[2] Mouttotou N, Green LE (1999). Incidence of foot and skin lesions in nursing piglets and their association with behavioural activities. Vet Rec.

[3] Kilbride AL, Mendl M, Statham P, Held S, Harris M, Cooper S (2012). A cohort study of preweaning piglet mortality and farrowing accommodation on 112 commercial pig farms in England. Prev Vet Med.

[4] Westin R, Holmgren N, Hultgren J, Ortman K, Linder A, Algers B (2015). Post-mortem findings and piglet mortality in relation to strategic use of straw at farrowing. Prev Vet Med.

[5] Gillman CE, KilBride AL, Ossent P, Green LE (2008). A cross-sectional study of the prevalence and associated risk factors for bursitis in weaner, grower and finisher pigs from 93 commercial farms in England. Prev Vet Med.

[6] Gillman CE, KilBride AL, Ossent P, Green LE (2009). A cross-sectional study of the prevalence of foot lesions in post-weaning pigs and risks associated with floor type on commercial farms in England. Prev Vet Med.

[7] Pluym LM, Van Nuffel A, Van Weyenberg S, Maes D (2013). Prevalence of lameness and claw lesions during different stages in the reproductive cycle of sows and the impact on reproduction results. Animal.

[8] Heinonen M, Peltoniemi O, Valros A (2013). Impact of lameness and claw lesions in sows on welfare, health and production. Livest Sci.

[9] Cador C, Pol F, Hamoniaux M, Dorenlor V, Eveno E, Guyomarc'h C (2014). Risk factors associated with leg disorders of gestating sows in different group-housing systems: a cross-sectional study in 108 farrow-to-finish farms in France. Prev Vet Med.

[10] Huneau-Salaün A, Bougeard S, Balaine L, Eono F, Eveno É, Guillermic M (2021). Do rubber floor mats prevent lameness in gestating sows housed in large groups? A field experiment on three commercial farms in France. Animals.

[11] Bos E-J, Maes D, van Riet MMJ, Millet S, Ampe B, Janssens GPJ (2016). Locomotion disorders and skin and claw lesions in gestating sows housed in dynamic versus static groups. PLoS ONE.

[12] Gjein H, Larssen RB (1995). The effect of claw lesions and claw infections on lameness in loose housing of pregnant sows. Acta Vet Scand.

[13] Jørgensen B (2003). Influence of floor type and stocking density on leg weakness, osteochondrosis and claw disorders in slaughter pigs. Anim Sci.

[14] Penny RHC, Edwards MJ, Mulley R (1971). Clinical observations of necrosis of the skin of suckling piglets. Aust Vet J.

[15] Mouttotou N, Hatchell FM, Green LE (1999). The prevalence and risk factors associated with forelimb skin abrasions and sole bruising in preweaning piglets. Prev Vet Med.

[16] Zoric M, Sjölund M, Persson M, Nilsson E, Lundeheim N, Wallgren P (2004). Lameness in piglets. Abrasions in nursing piglets and transfer of protection towards infections with Streptococci from sow to offspring. J Vet Med B Infect Dis Vet Public Health.

[17] Verhovsek D, Troxler J, Baumgartner J (2007). Peripartal behaviour and teat lesions of sows in farrowing crates and in a loose-housing system. Anim Welf.

[18] KilBride AL, Gillman CE, Ossent P, Green LE (2009). A cross sectional study of prevalence, risk factors, population attributable fractions and pathology for foot and limb lesions in preweaning piglets on commercial farms in England. BMC Vet Res.

[19] Baumgartner J, Winkler U, Kofler J, Tichy A, Troxler J (2012). Claw lesions of piglets kept in different types of farrowing pens. Wiener Tieraerztl Monatsschr.

[20] Quinn AJ, Boyle LA, KilBride AL, Green LE (2015). A cross-sectional study on the prevalence and risk factors for foot and limb lesions in piglets on commercial farms in Ireland. Prev Vet Med.

[21] Gravås L (1979). Behavioural and physical effects of flooring on piglets and sows. Appl Anim Ethol.

[22] Smith WJ, Mitchell CD (1976). Floor surface treatment to prevent lameness in suckling piglets. Farm Build Prog.

[23] Svendsen J, Olsson O, Nilsson C (1979). The occurrence of leg injuries on piglets with the various treatment of the floor surface of the farrowing pen. Nord Vet Med.

[24] Zoric M, Nilsson E, Mattsson S, Lundeheim N, Wallgren P (2008). Abrasions and lameness in piglets born in different farrowing systems with different types of floor. Acta Vet Scand.

[25] Bragulla H (1991). Die hinfällige Hufkapsel (*Capsula ungulae* decidua) des Pferdefetus und neugeborenen Fohlens*. Anat Histol Embryol.

[26] Westin R, Holmgren N, Hultgren J, Algers B (2014). Large quantities of straw at farrowing prevents bruising and increases weight gain in piglets. Preve Vet Med.

[27] Furniss SJ, Edwards SA, Lightfoot AL, Spechter HH (1986). The effect of floor type in farrowing pens on pig injury. I. Leg and teat damage of suckling piglets. Br Vet J.

[28] Zoric M, Nilsson E, Lundeheim N, Wallgren P (2009). Incidence of lameness and abrasions in piglets in identical farrowing pens with four different types of floor. Acta Vet Scand.

[29] Gundlach H (2010). Brutfürsorge, Brutpflege, Verhaltensontogenese und Tagesperiodik beim Europäischen Wildschwein (*Sus scrofa* L.)1. Z Tierpsychol.

[30] Reiner G, Kuehling J, Loewenstein F, Lechner M, Becker S (2021). Swine inflammation and necrosis syndrome (SINS). Animals.

[31] Reiner G, Lechner M, Eisenack A, Kallenbach K, Rau K, Muller S (2019). Prevalence of an inflammation and necrosis syndrome in suckling piglets. Animal.

[32] Reiner G, Kühling J, Lechner M, Schrade H, Saltzmann J, Muelling C (2020). Swine inflammation and necrosis syndrome is influenced by husbandry and quality of sow in suckling piglets, weaners and fattening pigs. Porcine Health Manag.

[33] Reiner G (2019). Entzündungs- und Nekrosesyndrom beim Schwein (SINS). Dtsch Tieraerzteblatt.

[34] Kuehling J, Loewenstein F, Wenisch S, Kressin M, Herden C, Lechner M (2021). An in-depth diagnostic exploration of an inflammation and necrosis syndrome in a population of newborn piglets. Animal.

[35] Seiferle E, Frewein, J. Aktiver Bewegungsapparat, Muskelsystem, Myologie - Statik und Dynamik des Bewegungsapparates. Nickel, R ,Schummer, A, Seiferle, E, Lehrbuch der Anatomie der Haustiere, Bd 1, Bewegungsapparat, 6 Auflage, S, 556. 1992.

[36] Hartsock TG, Graves HB (1976). Neonatal behavior and nutrition-related mortality in domestic swine. J Anim Sci.

[37] Alvåsen K, Hansson H, Emanuelson U, Westin R (2017). Animal welfare and economic aspects of using nurse sows in Swedish pig production. Front Vet Sci.

[38] Falke A, Friedli K, Gygax L, Wechsler B, Sidler X, Weber R (2018). Effect of rubber mats and perforation in the lying area on claw and limb lesions of fattening pigs. Animal.

[39] Geiping L, Hartmann M, Kreienbrock L, Grosse-Beilage E (2022). Killing underweighted low viable newborn piglets: Which health parameters are appropriate to make a decision?. Porcine Health Manag.

[40] Veit C, Traulsen I, Hasler M, Tölle K-H, Burfeind O, Beilage EG (2016). Influence of raw material on the occurrence of tail-biting in undocked pigs. Livest Sci.

[41] Hälli O, Haimi-Hakala M, Oliviero C, Heinonen M (2020). Herd-level risk factors for chronic pleurisy in finishing pigs: a case-control study. Porcine Health Manag.

[42] De Passille AMB, Rushen J (1989). Suckling and teat disputes by neonatal piglets. Appl Anim Behav Sci.

[43] Smith WJ (1979). Foot and limb disorders in baby piglets. Pig J.

[44] Penny RHC (1979). Genetical, physiological and anatomic factors contributing to foot and limb disorders in growing and adult pigs including a statistical review of foot and limb disorders in pigs attributable to floors. Pig J.

[45] Webb NG, Penny RHC, Johnston AM (1984). Effect of a dietary supplement of biotin on pig hoof horn strength and hardness. Vet Rec.

[46] Kuehling J, Eisenhofer K, Lechner M, Becker S, Willems H, Reiner G (2021). The effects of boar on susceptibility to swine inflammation and necrosis syndrome in piglets. Porcine Health Manag.

[47] Lechner M, Reiner G. SINS. Nutztierpraxis Aktuell. 2017;56/2017:14-6.

