# Supplementary material for: Foot lesions and forelimb skin abrasions in suckling piglets: development and risk factors
Source: Porcine Health Manag. 2024 Jan 4;10:1. doi: 10.1186/s40813-023-00351-9 (PMC10768078; doi:10.1186/s40813-023-00351-9)
Supplement: Supplementary file 1 — Additional file 1: Removal of capsula ungulae decidua in a newborn piglet. [file 40813_2023_351_MOESM1_ESM.docx]

**Additional file 1** Removal of *capsula ungulae decidua* in a newborn piglet

| Time after birth | Claw | Heel |
| --- | --- | --- |
| 00:00 h | 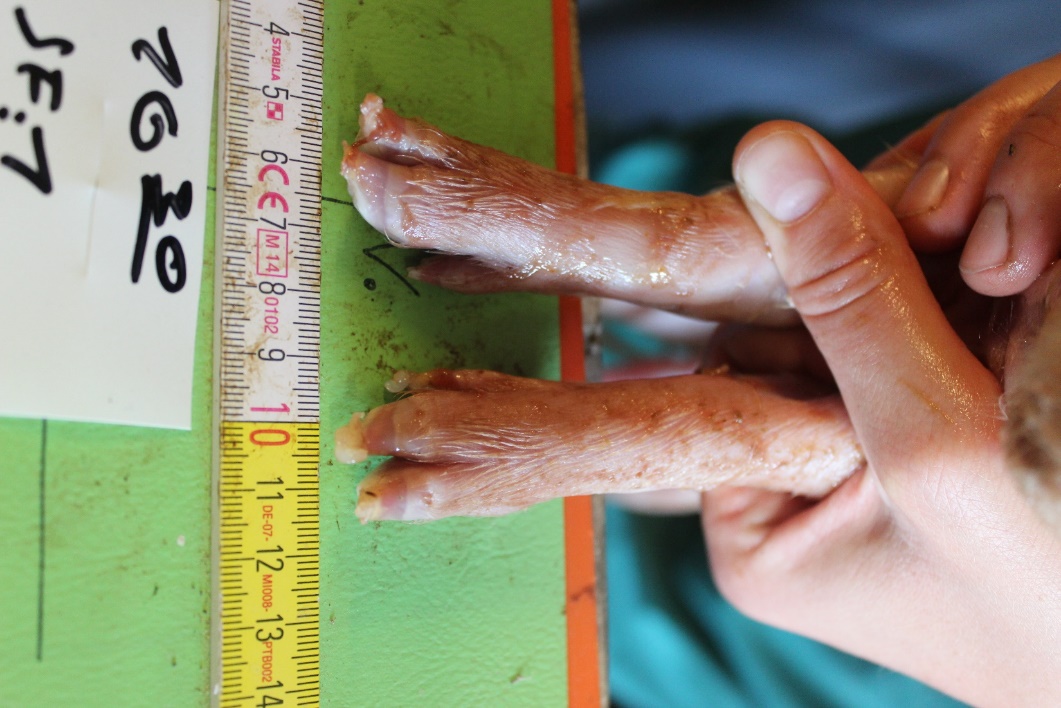 | 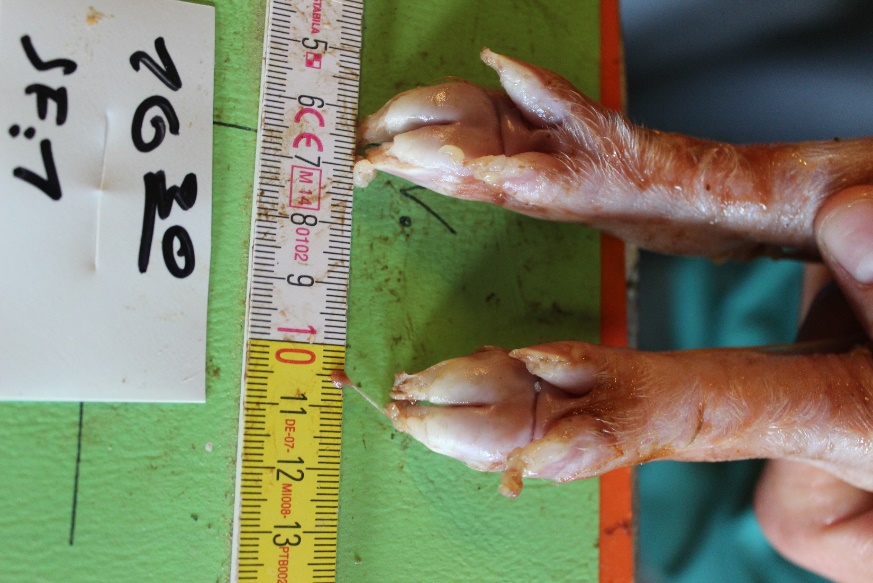 |
| + 01:00 h | 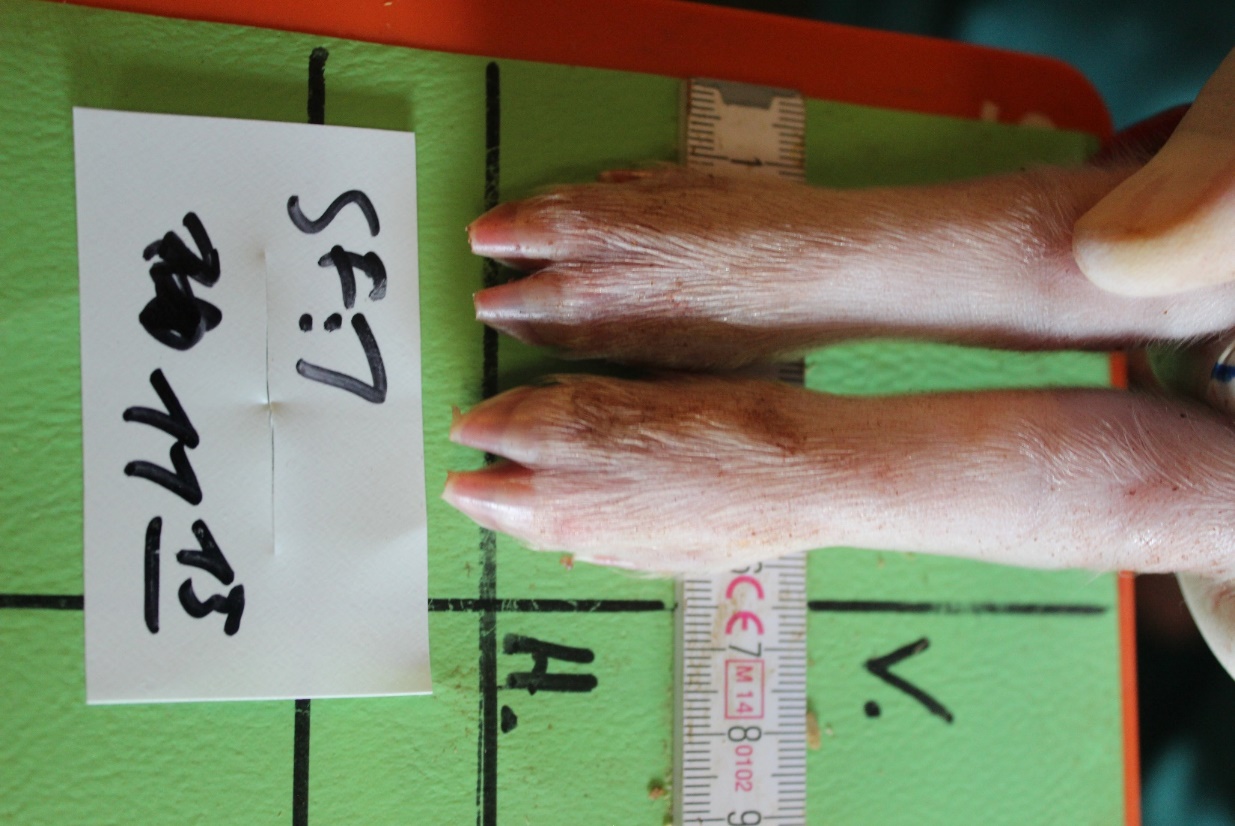 | 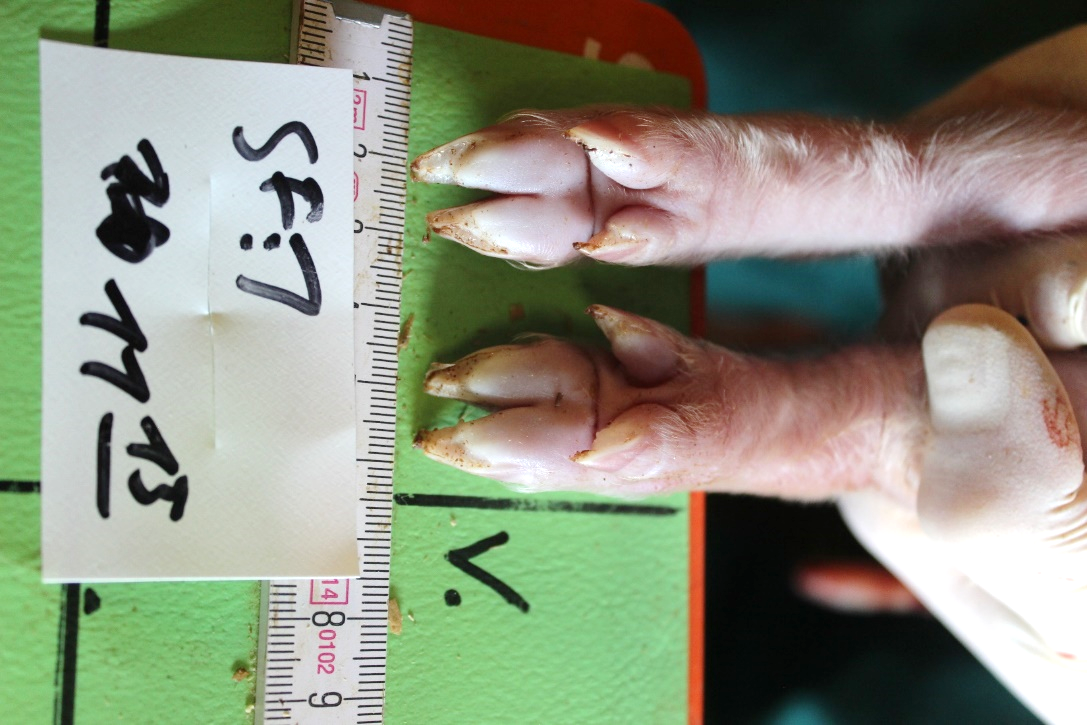 |
| +02:35 h | 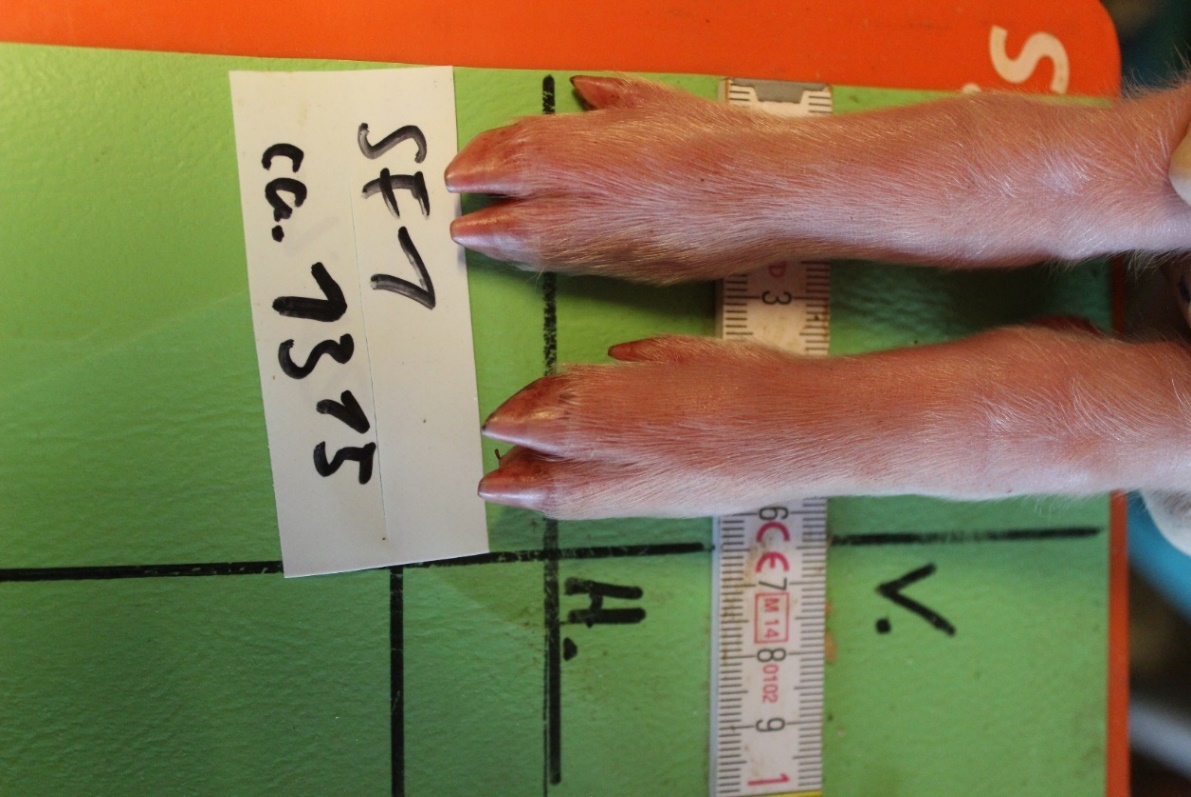 | 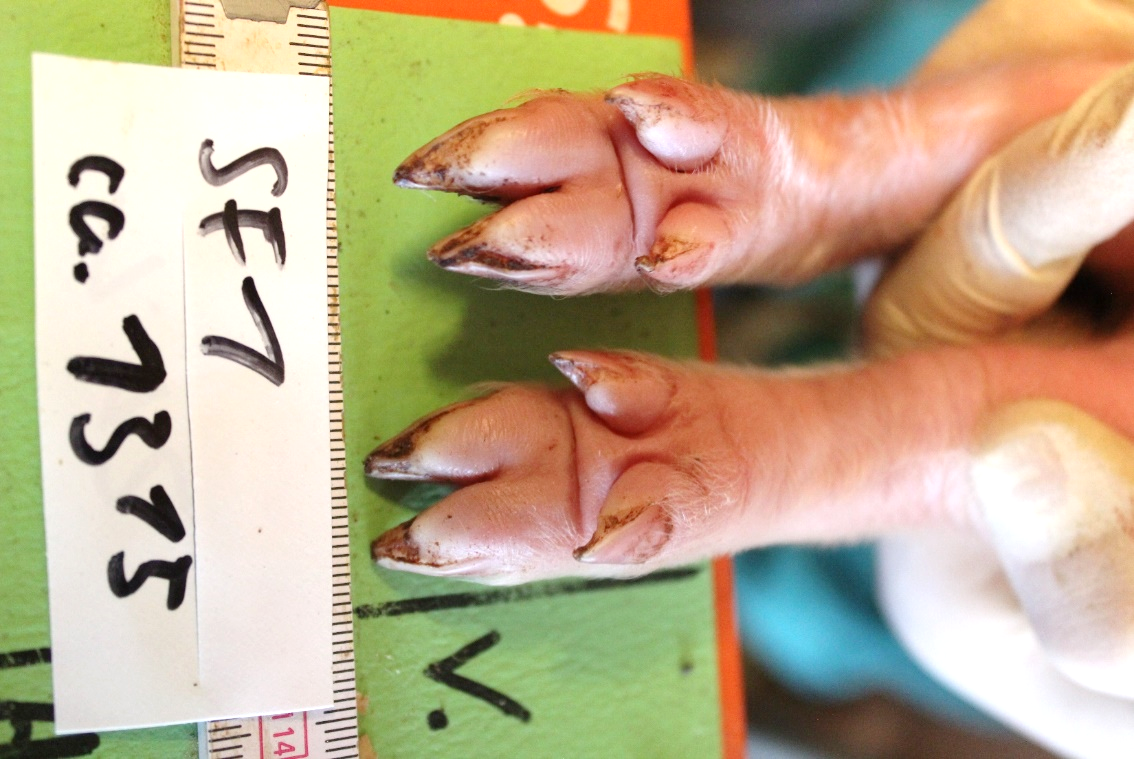 |
