# Supplementary material for: Foot lesions and forelimb skin abrasions in suckling piglets: development and risk factors
Source: Porcine Health Manag. 2024 Jan 4;10:1. doi: 10.1186/s40813-023-00351-9 (PMC10768078; doi:10.1186/s40813-023-00351-9)
Supplement: Supplementary file 2 — Additional file 2: Variation in suckling-postures of new-born piglets lead to contact of different localisations of fore and hint limbs with the floor. [file 40813_2023_351_MOESM2_ESM.docx]

**Additional file 2** Variation in suckling-postures of newborn piglets lead to contact of different localisations of fore and hint limbs with the floor (arrows indicate contact of coronary band with the floor)

| a) | b) |
| --- | --- |
| 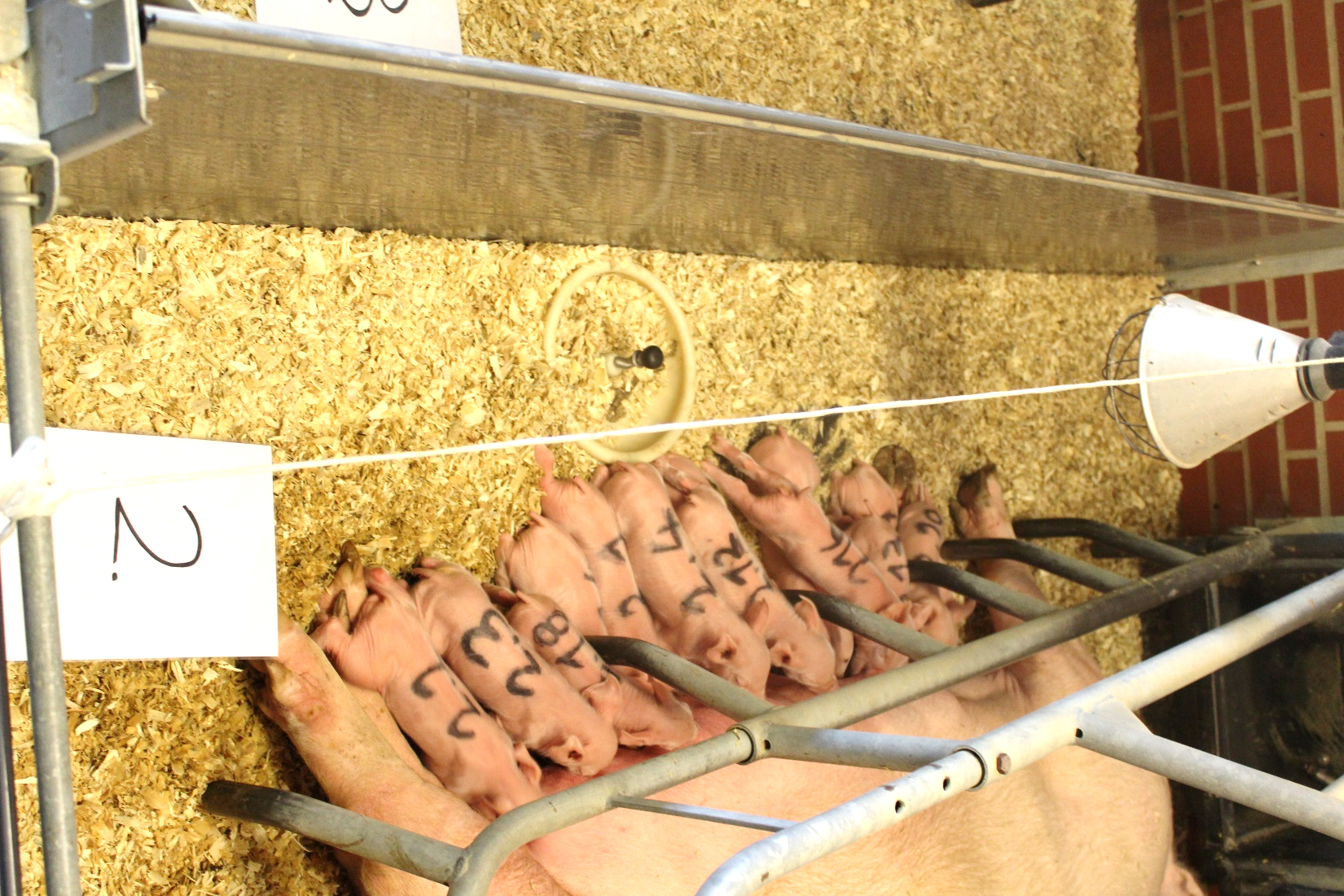 | 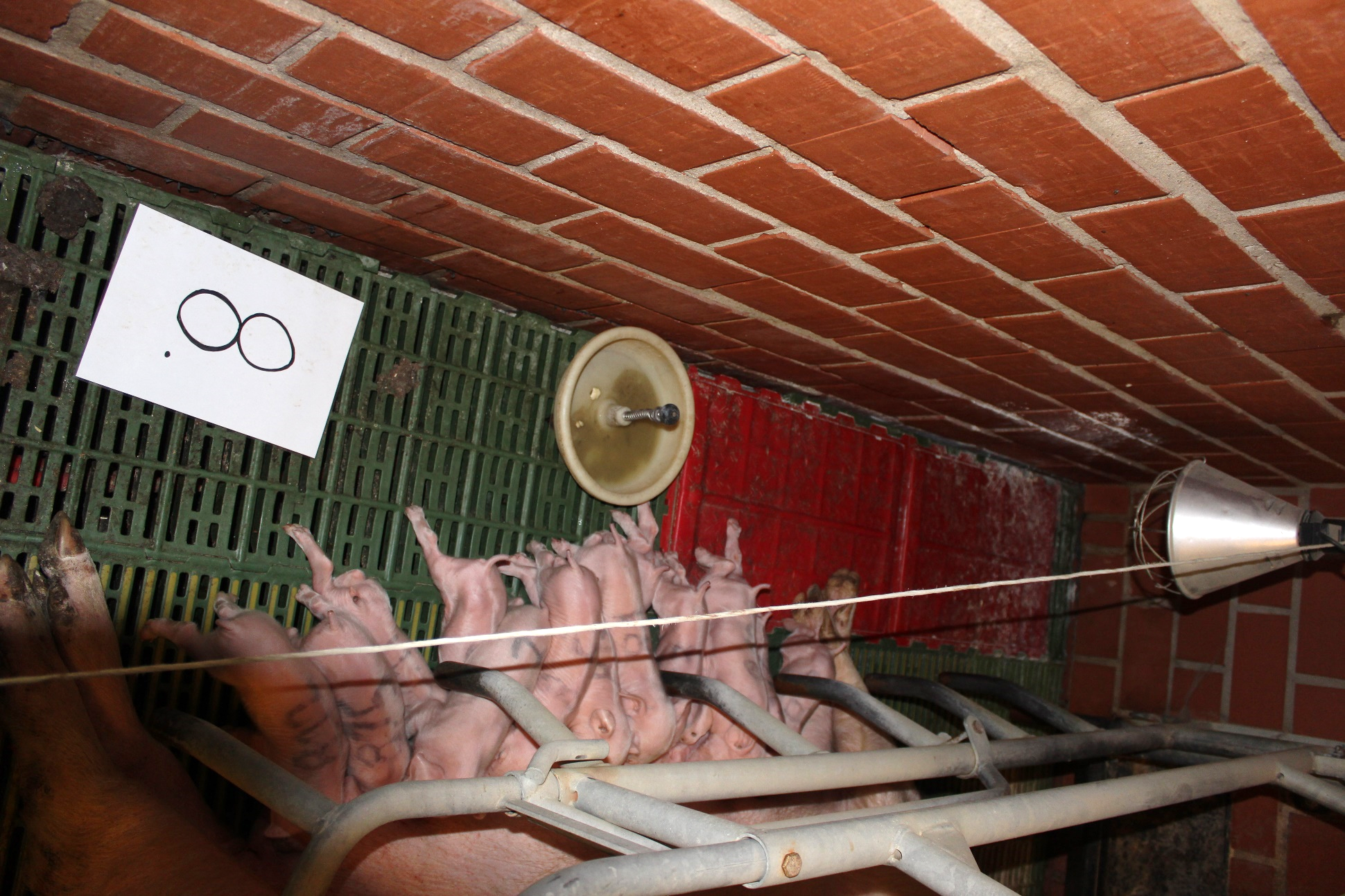 |
| c) | |
| 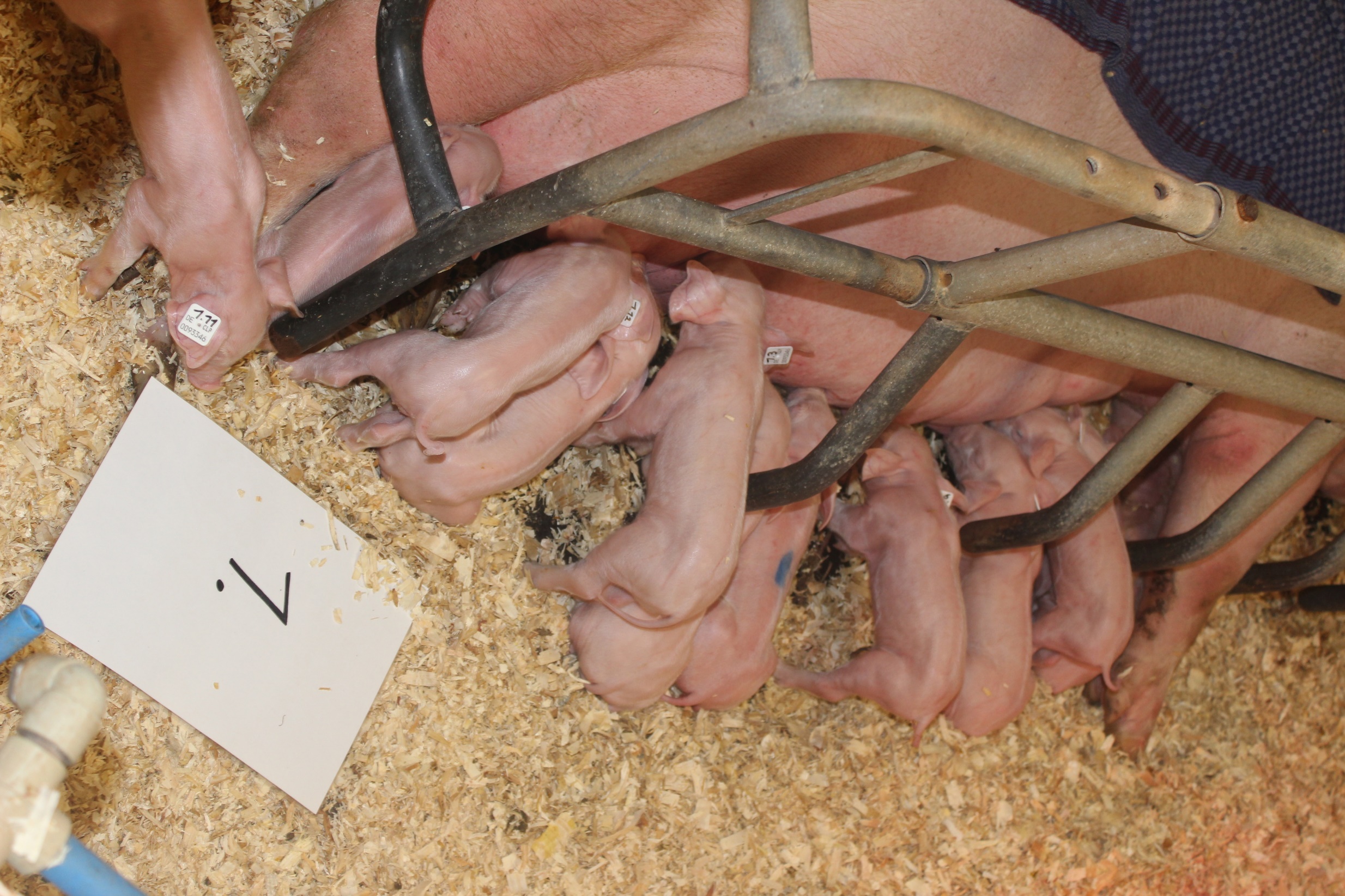 | |
